# QTL mapping and identification of SNP-haplotypes affecting yield components of *Theobroma cacao* L.

**DOI:** 10.1038/s41438-020-0250-3

**Published:** 2020-03-01

**Authors:** Luciel dos Santos Fernandes, Fábio M. Correa, Keith T. Ingram, Alex-Alan Furtado de Almeida, Stefan Royaert

**Affiliations:** 1 Mars Center for Cocoa Science, CP 55, Itajuípe, BA CEP 45.630-000 Brazil; 2 Departamento de Ciências Biológicas, Universidade Estadual de Santa Cruz, Rodovia Ilhéus-Itabuna, Km 16, Bairro Salobrinho, Ilhéus, BA CEP 45.662-900 Brazil; 3 Mars, Incorporated, 13601 Old Cutler Road, Miami, FL 33158 USA

**Keywords:** Plant breeding, Genetic markers, Plant breeding, Plant physiology

## Abstract

Cacao is a crop of global relevance that faces constant demands for improved bean yield. However, little is known about the genomic regions controlling the crop yield and genes involved in cacao bean filling. Hence, to identify the quantitative trait loci (QTL) associated with cacao yield and bean filling, we performed a QTL mapping in a segregating mapping population comprising 459 trees of a cross between ‘TSH 1188’ and ‘CCN 51’. All variables showed considerable phenotypic variation and had moderate to high heritability values. We identified 24 QTLs using a genetic linkage map that contains 3526 single nucleotide polymorphism (SNP) markers. Haplotype analysis at the significant QTL region on chromosome IV pointed to the alleles from the maternal parent, ‘TSH 1188’, as the ones that affect the cacao yield components the most. The recombination events identified within these QTL regions allowed us to identify candidate genes that may take part in the different steps of pod growth and bean filling. Such candidate genes seem to play a significant role in the source-to-sink transport of sugars and amino acids, and lipid metabolism, such as fatty acid production. The SNP markers mapped in our study are now being used to select potential high-yielding cacao varieties through marker-assisted selection in our existing cacao-breeding experiments.

## Introduction

Breeding programs for many crops focus on the improvement of crop yield components. Cacao yield components refer to the tree’s organs that are harvested and converted into final crop production, such as the total number of healthy pods, the fresh and dry bean weight, and the final yield. Besides resistance to biotic and abiotic stress and bean quality, yield (measured as dry bean weight in kilograms per hectare (kg/ha)) is also the most economically important crop trait, and it depends on a complex interaction between different factors that include genetics, environment, crop management, and growth and development processes^1^.

An essential step toward the selection of uniformly high-yielding cacao varieties is the identification of genomic regions and genes that control those yield components. A primary objective is to identify reliable molecular markers flanking the regions that control such traits. To date, few molecular markers have been associated with cacao yield components, such as pod traits and tree vigor^2^, and bean size and bean weight^3^, however, these studies did not report any candidate genes that control these traits. The association of candidate gene models with the genome-wide association and the mapping of QTL became possible after the publication of two cacao genomes, the Criollo genome^4^ and the Amelonado genome^5^. The expectation is to integrate the information from QTL regions to identify gene sequences controlling the phenotypic variation of traits of interest.

The genes that underpin many yield components exert regulatory control over biomass production and accumulation by acting in metabolic pathways that supply storage forms of nitrogen^6^, synthesize and transport carbon reserve compounds^7,8^ and lipids^9^ required during crop yield formation.

Little is known about the effects of genes that are involved in the transport of carbohydrates and in the synthesis of lipids during cacao bean filling. These genes may act in crucial stages of the reproductive and growth phases, such as the initial fertilization, pod set and further maintenance of pods, bean filling and seed germination. Just as they might be involved in the defense mechanisms against pathogens^10^, such genes might also be crucial to the processes involved in photoassimilate fluxes from source-to-sink organs. Although the physiological mechanisms have been explored in other crops, the genomic regions controlling those mechanisms were not studied in *T. cacao*.

To date, SNP-based QTL mapping of genomic regions associated with yield components has not been reported for *T. cacao*. Therefore, focusing on QTL mapping and on the identification of candidate genes affecting cacao yield components will provide an initial framework to understand the cacao yield formation. Moreover, this approach will allow us to investigate the inherent patterns of bean filling of each cacao genotype, and the identification of the regions that control such filling patterns is thereby an essential step in the direction of improving cacao yield.

The main objectives of this study were (1) to map QTL regions associated with cacao yield components (number of pods, pod index, dry bean weight and yield), (2) to identify candidate genes that have a higher probability of affecting the phenotypic variation of those traits, and (3) to provide reliable SNP markers to support the selection of high-yielding cacao genotypes via marker-assisted selection. In addition, we discuss the effect of significant recombination events affecting the phenotypic variation of the yield components and the candidate genes involved in the synthesis and the source-to-sink transport and accumulation of carbohydrates and amino acids, as well as in the breakdown of lipids to produce fatty acids in the cacao beans.

## Results

### Phenotypic correlations and multivariate analysis of cacao yield components

**Fig. 1:**
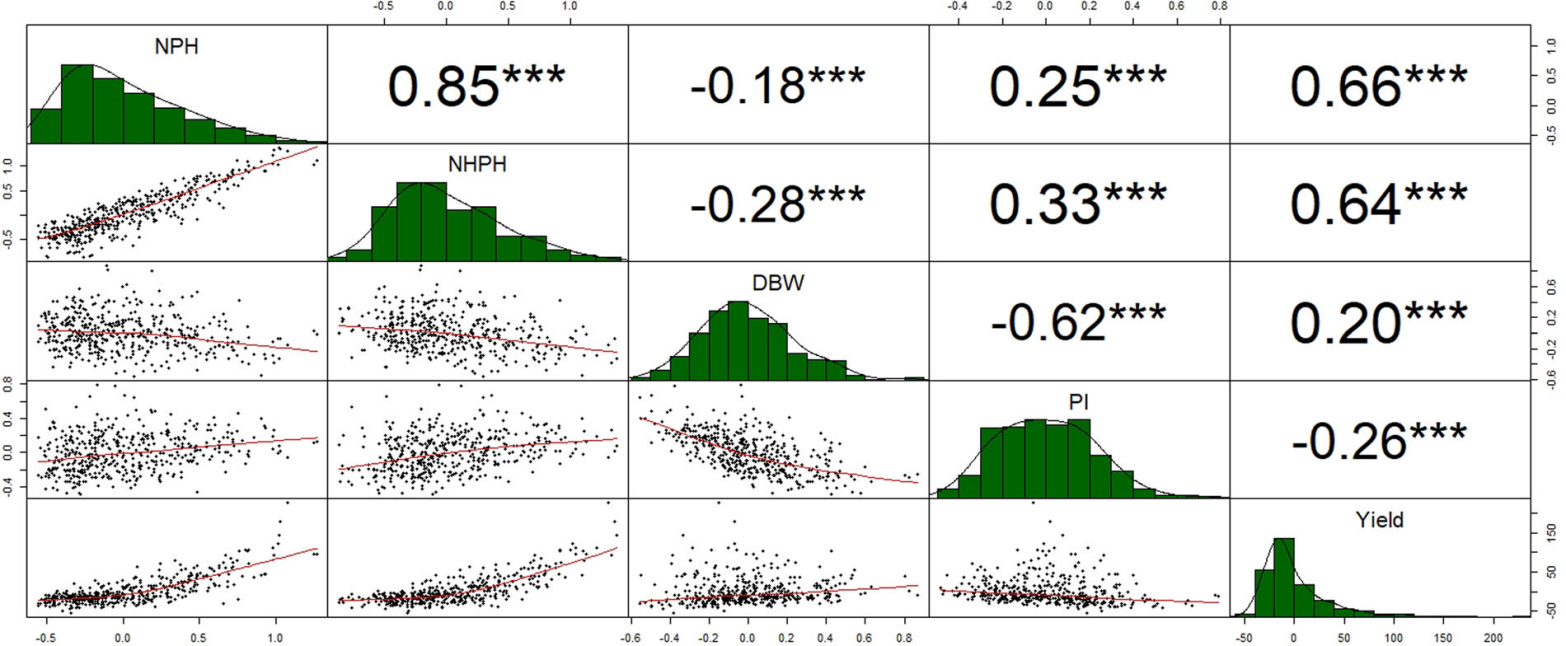
Spearman rank correlations (upper right), scatterplots (lower left), and histograms (diagonal) and for the yield components evaluated in the MP01 mapping population. Significance levels are ***p < 0.001; **p < 0.01; *p < 0.05. NPH is the total number of pods harvested; NHPH is the total number of healthy pods harvested; DBW is the dry bean weight of a single bean; PI is the pod index; Yield is the yield average

We computed the Spearman correlation among variables evaluated for 459 trees of MP01. Figure 1 shows the correlation coefficients (*r*) estimated for each pair of variables evaluated, as well as their relationships (scatterplots) and frequency distributions. Correlations for all variables evaluated were significant (*p*-value < 0.001), those among the strong correlations were between NPH and NHPH (*r* = 0.85), NPH and Yield (*r* = 0.66), NHPH and Yield (*r* = 0.64), and between DBW and PI (*r* = −0.64). Table 1 includes a summary of the phenotypic data and broad-sense heritability for the variables evaluated. In general, the MP01 population has high broad-sense heritability for cacao yield components. The pod-related variables, NPH and NHPH, had the highest heritability values of 0.73 and 0.75, respectively. Heritability values for PI and DBW were 0.62 and 0.56, respectively, while the average yield had a low heritability of 0.10.

**Table 1:** Description, summary statistics, and broad-sense heritability of cacao yield components evaluated in the MP01 mapping population

Variables	Description	Unit	Mean	SD	Min	Max	H^2^
NPH	Total number of pods harvested	number/tree	3.08	3.26	0	40	0.73
NHPH	Number of healthy pods harvested	number/tree	1.77	2.41	0	30	0.75
DBW	Dry bean weight of a single bean	g	1.47	0.35	0.37	3.26	0.56
PI	Pod index	number/tree	21.25	12.43	6	241	0.62
Yield	Yield average (dry bean weight)	kg/ha	144.83	143.55	0.00	1635.70	0.10

Broad-sense heritability (H^2^), and SD stands for standard deviation

**Fig. 2:**
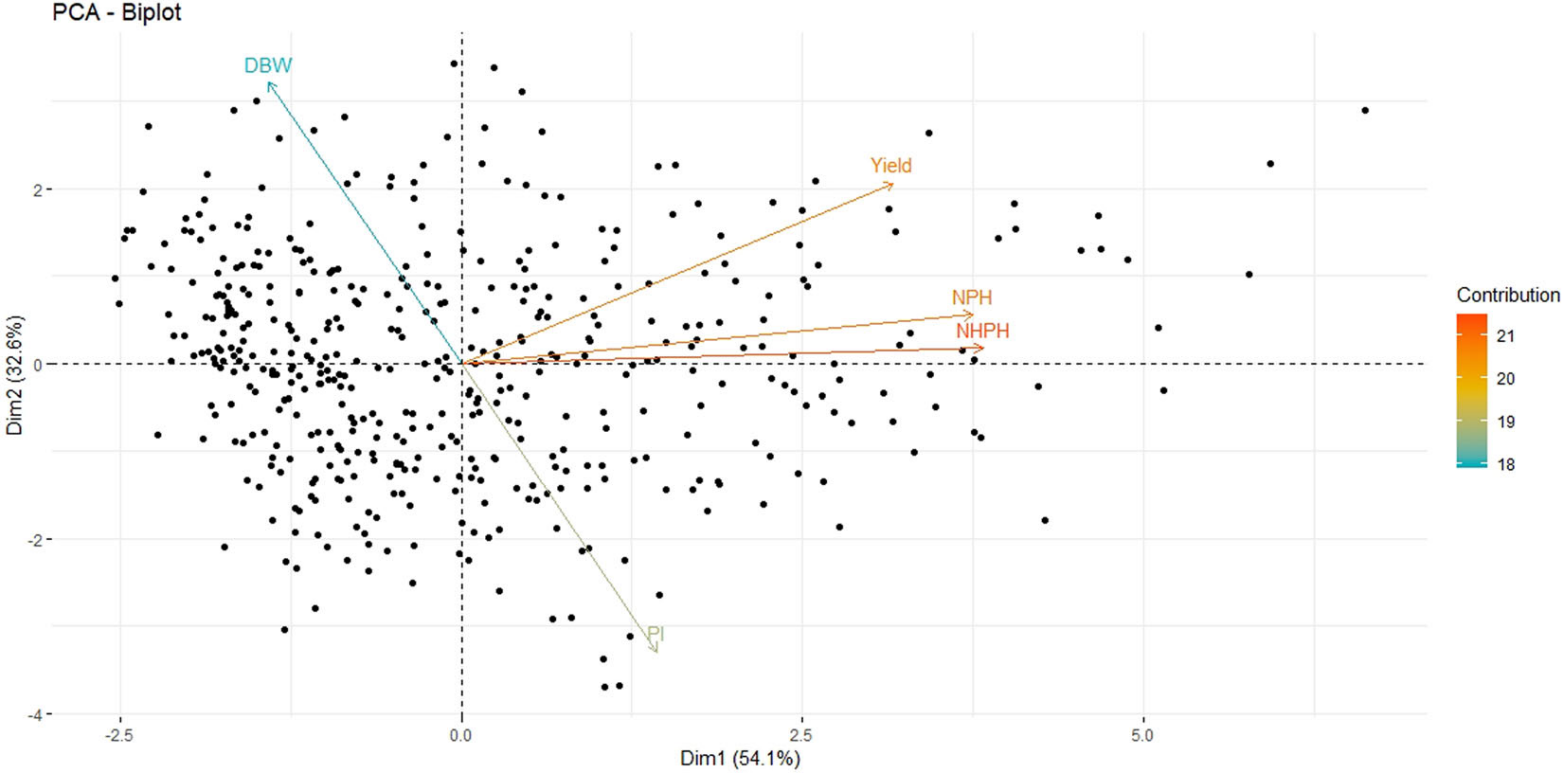
Biplot of the first and second principal components from phenotypic yield data from the MP01 mapping population. NPH = number of total pods harvested, Yield = average yield, DBW = dry bean weight of a single bean, and PI = pod index. Arrow length and direction indicate the association with a particular component, while the clustering of vectors indicates the correlation among variables

We present the amount of variation retained (eigenvalues) by the first two principal components for the MP01 individuals and yield components variables (Fig. 2, Supplementary Table 1 and S2). The first two principal components (Dim.1 and Dim.2) accounted for 86.91% of the cumulative phenotypic variation, in which the first principal component (Dim.1) retained 54.4%, while the second (Dim.2) retained 32.5% (Fig. 2). Together with the third component (Dim.3 = 7.8%), the principal components explained 94.77% of the cumulative phenotypic variations (Supplementary Table S3). The first principal component showed a contrast between DBW and the variables NPH, NHPH, PI, and Yield, which indicates a regulation of partitioning of assimilated carbon because of the increased number of harvestable organs at the plant level. The variables that positively contributed in the first principal component were NPH, NHPH and Yield (Supplementary Table 2), which are the main variables contributing in the phenotypic variation of the individuals and yield components data (Fig. 2). In the second principal component, the variables DBW and PI showed the highest and most contrasting eigenvalues, 0.81 and −0.83, respectively (Supplementary Table 2).

### Detection of QTLs associated with cacao yield components

**Fig. 3:**
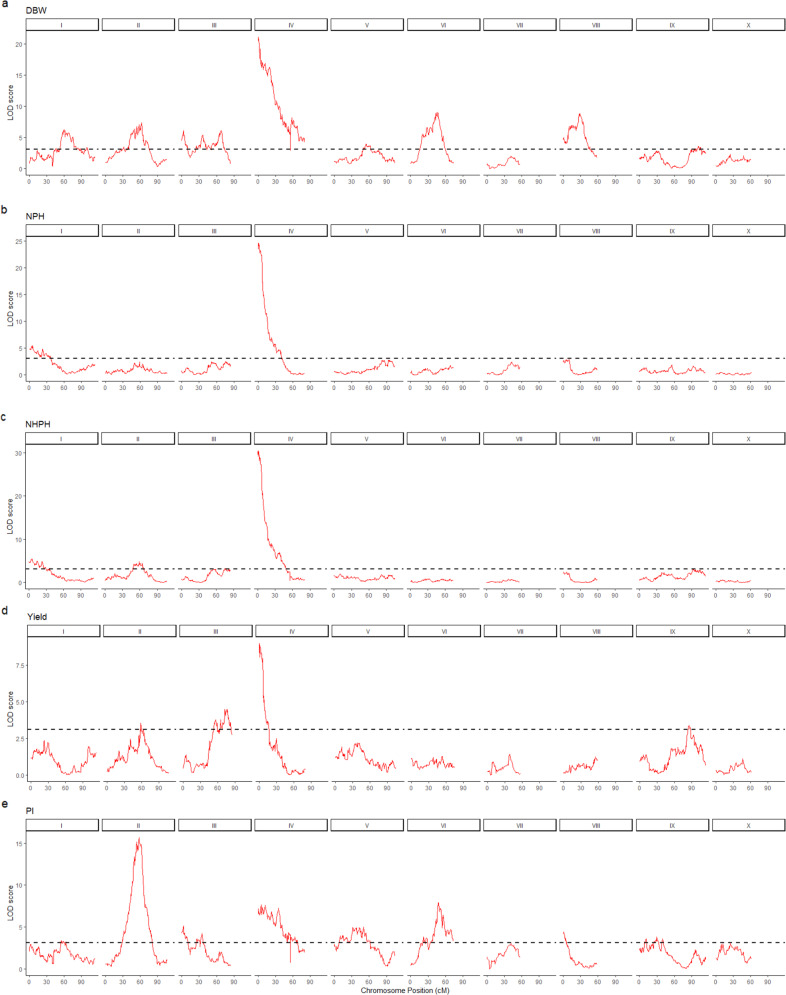
Positions of QTLs identified for the yield components identified in the mapping population MP01 (‘TSH 1188’ × ‘CCN 51’). On the top of each panel are the chromosome numbers, and the x-axis is showing the positions of the SNP markers in centimorgans (cM). The *y*-axis is presenting the logarithm of the odds (LOD) scores from the interval mapping analysis. The panels are presented as following: DBW (**a**), NPH (**b**), NHPH (**c**), Yield (**d**) and PI (**e**). The dotted line represents the LOD score threshold of 3.1 (*p*-values < 0.05)

The QTL intervals flanked by SNP markers with LOD scores higher than 3.1 were considered as significant. We used the genetic linkage map from the MP01 population (‘TSH 1188’ × ‘CCN 51’), published in 2016^11^. Figure 3 provides the genomic positions and Table 2 the percentage of phenotypic variation explained by each region flanked by the SNP markers with significant LOD scores. In total, we mapped 24 QTL positions across eight different chromosomes, except for the chromosomes VII and X.

**Table 2:** QTLs identified for the total number of pods harvested (NPH), the number of healthy pods harvested (NHPH), dry bean weight (DBW), pod index (PI), and average yield

Variable		Chr.	Parent	cM	LOD	% Expl.	TSH 1188		CCN 51	
							T1	T2	C1	C2
NPH	Tcm001s01308520	I	P2	5.38	4.99	3.8	C	T	C	T
	Tcm004s00615809	IV	P1	3.29	23.5	19.7	**A**	**G**	A	G
NHPH	Tcm001s01308520	I	P1	5.38	4.21	3.3	C	T	C	T
	Tcm004s00615809	IV	P2	3.29	28.02	23.6	**A**	**G**	A	G
DBW	Tcm001s18406546	I	P1	58.04	8.21	2.7	T	C	T	C
	Tcm002s07453904	II	P1	41.22	3.58	1.2	G	A	G	A
	Tcm002s15300388	II	P2	53.65	3.28	1.1	T	C	T	C
	Tcm003s00621672	III	P2	3.2	7.18	2.4	C	A	A	C
	Tcm003s27977985	III	P2	57.54	4.2	1.4	A	G	G	A
	Tcm003s30366194	III	P1	68.34	16.6	5.7	C	T	C	T
	Tcm004s00289192	IV	P1	0.11	37.59	14.6	**A**	**G**	A	G
	Tcm004s30466731	IV	P1	66.57	10.66	3.6	C	T	C	T
	Tcm005s29846151	V	P1	58.56	6.15	2	G	T	G	T
	Tcm005s33377306	V	P2	72.35	11.41	3.8	T	G	T	G
	Tcm005s38257511	V	P2	94.52	4.24	1.4	A	G	A	G
	Tcm006s20470604	VI	P2	33.86	12.33	4.2	C	A	C	A
	Tcm006s22739149	VI	P1	47.26	9.27	3.1	A	C	A	C
	Tcm008s04113686	VIII	P2	27.41	23.68	8.5	C	T	C	T
	Tcm009s03430458	IX	P1	21.71	8.59	2.9	C	T	C	T
	Tcm009s39845182	IX	P1	101.35	4.35	1.4	T	C	T	C
PI	Tcm001s18406546	I	P1	58.04	4.75	2.8	T	C	T	C
	Tcm002s07453904	II	P1	41.22	4.25	2.4	G	A	G	A
	Tcm002s23708704	II	P2	57.94	9.23	5.4	**G**	**A**	G	A
	Tcm003s00621672	III	P2	3.2	3.25	1.4	C	A	A	C
	Tcm003s29414854	III	P1	64.59	5.16	2.2	C	T	C	T
	Tcm004s01127580	IV	P1	5.16	7.1	4.1	**G**	**A**	A	G
	Tcm005s24838905	V	P1	44.45	4.18	2.4	G	A	A	G
	Tcm006s22739149	VI	P1	47.26	8.81	5.2	A	C	A	C
	Tcm008s00170802	VIII	P2	0.11	4.4	2.5	T	C	T	C
	Tcm009s39845182	IX	P1	101.35	3.41	3	T	C	T	C
Yield	Tcm003s29414854	III	P1	64.59	3.11	3.4	C	T	C	T
	Tcm004s00615809	IV	P1	3.3	7.97	7.2	**A**	**G**	A	G
	Tcm009s36667972	IX	P2	85.1	3.4	3	**C**	**T**	C	T

Markers with the highest LOD scores for each trait are indicated in bold. P1 and P2 are referring to which parent the QTL is from, ‘TSH 1188’ and ‘CCN 51’, respectively, according to the MQM mapping

Of those QTLs, we highlight a region at the top of chromosome IV that was flanked by three significant SNP markers; they are Tcm004s00289192, Tcm004s00615809, and Tcm004s01127580. Their LOD values ranged from 7.10 to 37.59, while the percentage of phenotypic variation ranged from 4.10 to 23.60%.

### Analysis of haplotype–phenotype associations

The haplotypes from the mother ‘TSH 1188’ are represented with “T”, and with “C” for the father, ‘CCN 51’. For each trait-marker association, we performed the variance analysis on the means of the four parental haplotype combinations (T1C1, T1C2, T1C2, and T2C2), which showed different levels of significance (*p* < 0.001, *p* < 0.01 and *p* < 0.05) (Table 3). There was no statistically significant difference between the haplotype groups for the markers Tcm003s29414854 for PI, Tcm005s38257511, and Tcm009s03430458 for DBW. Tukey’s test showed that the haplotype T2 on chromosome IV (Tcm004s00615809, *p* < 0.001) had a significant effect on increase the total number of pods harvested (NPH and NHPH). For variable DBW, the haplotype T1 showed a significant effect on also chromosome IV (Tcm004s00289192 and Tcm004s30466731, *p* < 0.001), while T2 was significant for the SNP markers Tcm001s18406546, Tcm003s30366194, and Tcm006s22739149 (*p* < 0.001). For the SNP markers Tcm002s07453904, Tcm002s15300388, and Tcm008s04113686, the haplotype C1 has a significant effect in increasing DBW (*p* < 0.001). For the other markers, Tukey’s test did not show a clear distinction between groups.

**Table 3:** Tukey post hoc test for multiple comparisons to identify the difference in the effect of the parental haplotype combinations on the cacao yield components

Variable	SNP name	Mean				Tukey groups				*P*-value
		T1:C1	T1:C2	T2:C1	T2:C2	T1:C1	T1:C2	T2:C1	T2:C2	
NPH	Tcm001s01308520	7.39	5.96	6.68	4.79	a	ab	a	b	<0.01
	Tcm004s00615809	4.40	3.82	8.76	7.87	b	b	a	a	<0.001
NHPH	Tcm001s01308520	4.83	3.90	4.36	3.26	a	ab	a	b	<0.01
	Tcm004s00615809	2.93	2.63	5.52	5.33	b	b	a	a	<0.001
DBW	Tcm001s18406546	1.45	1.38	1.52	1.56	bc	c	ab	a	<0.001
	Tcm002s07453904	1.57	1.46	1.51	1.41	a	bc	ab	c	<0.001
	Tcm002s15300388	1.58	1.45	1.52	1.40	a	bc	ab	c	<0.001
	Tcm003s00621672	1.51	1.50	1.55	1.37	a	a	a	b	<0.001
	Tcm003s27977985	1.41	1.47	1.53	1.54	b	ab	a	a	<0.01
	Tcm003s30366194	1.40	1.45	1.53	1.56	c	bc	ab	a	<0.001
	Tcm004s00289192	1.59	1.61	1.38	1.35	a	a	b	b	<0.001
	Tcm004s30466731	1.55	1.56	1.43	1.42	a	a	b	b	<0.001
	Tcm005s29846151	1.49	1.56	1.42	1.48	ab	a	b	ab	<0.01
	Tcm005s33377306	1.48	1.55	1.43	1.48	ab	a	b	ab	<0.001
	Tcm005s38257511	1.55	1.49	1.45	1.48	a	ab	b	ab	0.073
	Tcm006s20470604	1.50	1.37	1.55	1.52	a	b	a	a	<0.001
	Tcm006s22739149	1.39	1.45	1.50	1.61	c	bc	b	a	<0.001
	Tcm008s04113686	1.40	1.54	1.44	1.59	b	a	b	a	<0.001
	Tcm009s03430458	1.53	1.51	1.46	1.45	a	a	a	a	0.062
	Tcm009s39845182	1.40	1.52	1.51	1.53	b	a	a	a	<0.001
PI	Tcm001s18406546	21.53	20.49	19.46	18.62	a	ab	b	b	<0.01
	Tcm002s07453904	18.14	19.98	19.44	21.97	b	b	b	a	<0.001
	Tcm002s23708704	17.68	19.92	19.21	22.87	c	b	bc	a	<0.001
	Tcm003s00621672	19.64	20.53	18.28	21.29	ab	a	b	a	<0.01
	Tcm003s29414854	20.74	20.24	19.24	19.35	a	a	a	a	0.188
	Tcm004s01127580	19.24	18.40	21.13	21.14	ab	b	a	a	<0.001
	Tcm005s24838905	18.95	18.75	20.78	21.27	bc	c	ab	a	<0.01
	Tcm006s22739149	21.13	20.90	19.09	18.50	a	ab	bc	c	<0.01
	Tcm008s00170802	21.19	19.19	20.52	18.67	a	ab	ab	b	<0.01
	Tcm009s39845182	21.34	19.34	18.93	19.85	a	ab	b	ab	<0.05
Yield	Tcm003s29414854	235.30	246.62	322.09	308.08	b	ab	a	a	<0.01
	Tcm004s00615809	222.86	195.62	361.34	337.28	b	b	a	a	<0.001
	Tcm009s36667972	326.57	243.07	287.49	238.24	a	b	ab	b	<0.01

The markers from the mother are also the ones that affect the pod index (PI) for the majority of the QTL regions. T1 was significant for the marker Tcm001s18406546, Tcm006s22739149, Tcm008s00170802, and Tcm009s39845182. The marker with higher LOD score for PI, Tcm002s23708704, the haplotype C2 from the ‘CCN 51’ was associated with an increased pod index. Finally, the T2 for the marker Tcm004s00615809 (*p* < 0.001) is the one associated with increased yield (Table 3).

**Fig. 4:**
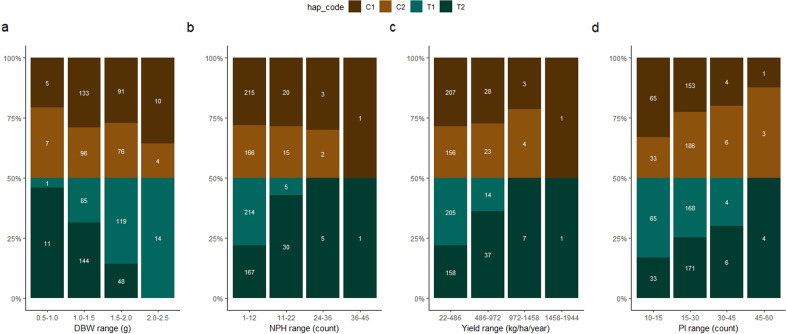
Haplotype frequencies for different phenotypical ranges of cacao yield components. The haplotype frequencies are presented in panels (**a**) to (**d**). The phenotypic ranges of cacao yield components are shown on the *x*-axis, while the *y*-axis shows the percentages for each parental haplotype. Panels (**a**), (**b**), and (**c**) are for the main SNP markers on chromosome IV (Tcm004s00289192, Tcm004s00615809, and Tcm004s01127580), mapped for dry bean weight (**a**), the total number of pods harvested (**b**), and average yield (**c**). Panel (**d**) is for the main SNP marker (Tcm002s23708704) for pod index on chromosome II

**Fig. 5:**
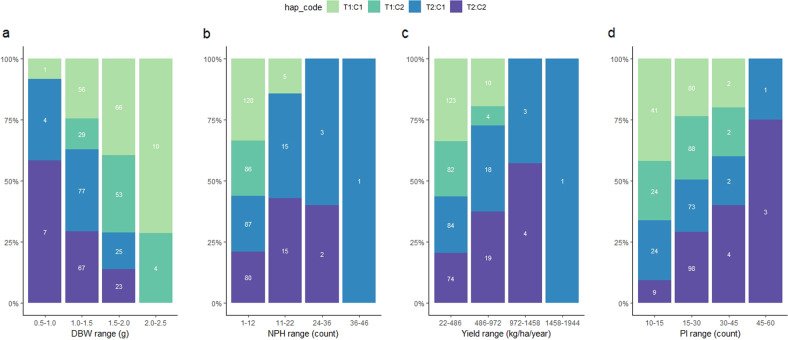
Haplotype combination frequencies for different phenotypical ranges of cacao yield components. The haplotypes frequencies are presented in panels (**a**) to (**d**). The different phenotypical ranges of cacao yield components are shown on the *x*-axis, while the *y*-axis shows the percentages of each parental haplotype. Panels (**a**), (**b**), and (**c**) are for the main SNP markers on chromosome IV (Tcm004s00289192, Tcm004s00615809, and Tcm004s01127580), mapped for dry bean weight (**a**), the total number of pods harvested (**b**), and average yield (**c**). Panel (**d**) is for the main SNP marker (Tcm002s23708704) for pod index on chromosome II

Although we mapped multiple significant QTLs linked to multi-variables of cacao yield, we focused on the main QTL regions mapped on chromosome IV, flanked by the markers Tcm004s00289192, Tcm004s00615809, and Tcm004s01127580 (for NPH, NHPH, DBW, PI, and Yield), and on chromosome II with the marker Tcm002s23708704 (only for PI). Those QTL regions were selected because the markers on chromosome IV showed a higher LOD score for all variables evaluated, and Tcm002s23708704 showed LOD score of 9.23, the highest for PI. The distance from Tcm004s00289192 to Tcm004s00615809 is 326.6 kbp, while Tcm004s00615809 is 511.8 kbp away from Tcm004s01127580. For analysis of haplotype–phenotype associations, we considered the interval from Tcm004s00289192 to Tcm004s01127580 as a unique haplotype. For that, each variable evaluated was classified into four phenotypical classes, into which we counted the haplotype frequency of each parental haplotype (T1, T2, C1, and C2; Fig. 4) and their combinations (T1:C1, T1:C2, T2:C1, T2:C2, Fig. 5). The alleles from ‘TSH 1188’ (T1 and T2) were the most significantly associated with yield components (*p* < 0.01, Table 4, Figs. 4 and 5). The frequency of the T1 allele, which corresponds with SNP alleles AAG, increases along with a rise in DBW (Fig. 5a). On the other hand, the haplotype T2 (GGA) was associated with an increase in NPH, and yield (Fig. 5b, c). The same pattern observed for the marker Tcm002s23708704, in which the haplotype T2 (A) is associated with higher PI. Those results reflect the negative correlation between DBW and all the other variables. Therefore, the maternal parent, ‘TSH 1188’, is the one conferring the QTL on those chromosome regions.

**Table 4:** Associations between the parental haplotypes (T1, T2, C1, and C2) on chromosomes IV (Tcm004s00289192, Tcm004s00615809, and Tcm004s01127580) and II (Tcm002s23708704) and the relative frequency for each phenotypical class evaluated

Variable	Chr.	SNP	allele	Phenotypical ranges				*P*-value
				1–3	3–5	5–7	7–10	
**NPH**	IV	AAG	T1	0.32	0.17	0.13	0.00	7.18E−76
		**GGA**	T2	0.18	0.33	0.37	0.50	2.13E−21
		AAA	C1	0.28	0.31	0.30	0.21	1.23E−45
		GGG	C2	0.22	0.19	0.20	0.29	1.01E−38
				0.5–1.0	1.0–1.5	1.5–2.0	2.0–2.5	
**DBW**	IV	**AAG**	T1	0.05	0.19	0.35	0.50	1.77E−37
		GGA	T2	0.45	0.31	0.15	0.00	1.30E−52
		AAA	C1	0.23	0.29	0.28	0.30	2.22E−42
		GGG	C2	0.27	0.21	0.22	0.20	1.98E−30
				22–486	486–972	972–1458	1458–1944	
**Yield**	IV	AAG	T1	0.28	0.14	–	–	3.34E−126
		**GGA**	T2	0.21	0.38	0.44	0.50	4.71E−70
		AAA	C1	0.29	0.26	0.19	0.50	3.52E−116
		GGG	C2	0.21	0.21	0.31	–	8.70E−81
				11–23	23–35	35–47	47–60	
**PI**	IV	**AAG**	T1	0.29	0.18	0.25	0.50	1.08E−76
		GGA	T2	0.21	0.32	0.25	0.00	3.70E−47
		AAA	C1	0.28	0.30	0.38	0.25	8.57E−64
		GGG	C2	0.22	0.20	0.13	0.25	8.71E−55
				11–23	23–35	35–47	47–60	
**PI**	II	G	T1	0.29	0.22	0.13	0.00	3.11E−74
		**A**	T2	0.21	0.28	0.38	0.50	1.71E−46
		G	C1	0.29	0.15	0.25	0.00	5.20E−81
		A	C2	0.21	0.35	0.25	0.50	1.15E−46

Phenotypical ranges were defined based on the average data for each variable

### Identification of recombinant events and candidate genes

**Fig. 6:**
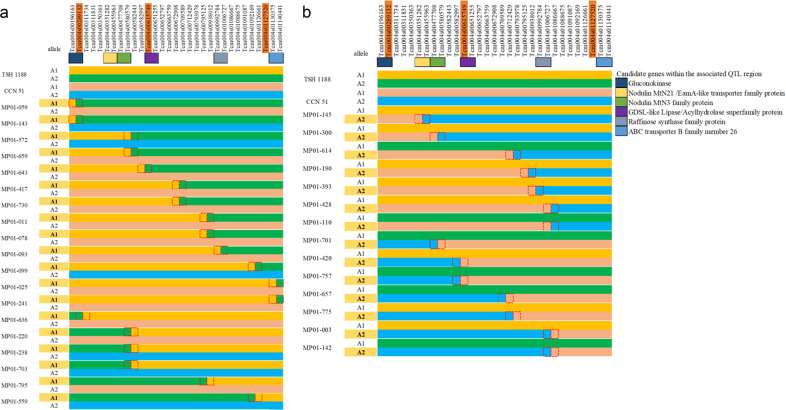
Haplotype analysis of trees exhibiting recombination events in the main QTL region on chromosome IV. **a** Trees showing recombination events between the maternal haplotypes, **b** trees showing recombination events between the paternal haplotypes. Maternal and paternal haplotypes are shown at the top of each panel. We highlighted the position where the recombination events are occurring. Boxes indicate the locations of the candidate genes at the top of each panel

To identify candidate genes, we examined trees exhibiting recombination events for both parental haplotypes within this region and between flanking SNP markers. We identified 33 trees displaying recombination events in at least one parental haplotype (Fig. 6a, b). Of those trees, 19 trees possessed recombination events between the maternal haplotypes (Fig. 6a) in an interval from 196,163 to 1,140,441 bp, while another 14 trees had a recombination event between the paternal haplotypes within the interval from 351,282 to 1,086,667 bp (Fig. 6b). Within this region, we identified recombination events occurring in ten different genomic positions, but with some recombination events occurring at the same spots.

**Fig. 7:**
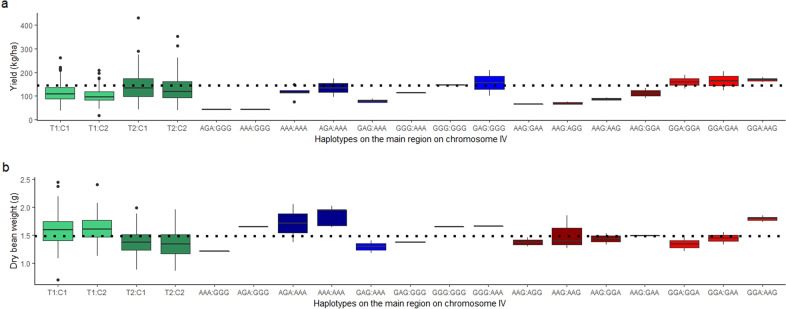
Boxplot of yield (**a**) and dry bean weight (**b**) data per haplotype in the main QTL region on chromosome IV. The trees with recombination events within the region are colored according to the parental haplotype where the recombination event occurred: non-recombinants AAG:AAA (T1:C1) and AAG:GGG (T1:C2) are colored in green; non-recombinants GGA:AAA (T2:C1) and GGA:GGG (T2:C2) are colored in dark green. Trees possessing recombination events on the maternal haplotype T1 (AGA:GGG, AAA:GGG, AAA:AAA, and AGA:AAA) are colored in dark blue. Trees possessing recombination events on the maternal haplotype T2 (GAG:AAA, GGG:AAA, GGG:GGG, and GAG:GGG) are colored in blue. Recombination events on paternal haplotype C1 (AAG:GAA, AAG:AGG, AAG:GGA, and AAG:AAG) are colored in dark red, and recombination events on C2 (GGA:GGA, GGA:GAA, and GGA:AAG) are colored in red. The dotted line represents the population average for yield and dry bean weight, respectively

To evaluate the effect of those recombination events, we calculated the average yield and dry bean weight for each haplotype combination and the different recombination events (Fig. 7a, b). The first three SNPs alleles represent the maternal haplotype, separated by colons from the three alleles of the paternal haplotype (e.g., AAG:AAA, maternal and paternal alleles, respectively). We highlighted in bold the position of each recombination event. The MP01 recombinants in which the maternal haplotype switched from T1 to T2 showed a lower average yield (84.33 kg/ha) than the population average (144.85 kg/ha). Those trees possessed the haplotypes A**GA**:GGG, A**GA**:AAA, AA**A**:GGG, and AA**A**:AAA. The average yield was of 83.49 kg/ha in the other recombinant trees possessing the T1 haplotype but combined with a recombination event on the paternal haplotype. Those recombinant haplotypes are AAG:G**AA**, AAG:GG**A**, and AAG:AA**G** (Fig. 7a).

On the other hand, we observed an average yield of 123.85 kg/ha in the recombinant trees in which the maternal haplotype switched from T2 to T1, and in the ones in which the maternal haplotype T2 is combined with a recombinant haplotype from the paternal parent (Fig. 7a). For those recombinant trees the average yield was 164.62 kg/ha, which is 13% more than the population average.

Those results confirm that the haplotype T2 (GGA) indeed is the favorable one to increase the yield in MP01 population. In contrast, trees possessing the haplotype T1 (AAG) showed higher dry bean weight compared with the others (Fig. 7b). The dry bean weight was 1.60 g in trees with recombination events on the haplotype T1 (Fig. 7b), while for the MP01 recombinants on the T2 haplotype was 1.49 g.

Based on the examination of the trees with recombination events between the maternal haplotypes, we delimited the region regulating the phenotypic variation of yield components from 196,163 to 1,140,441 (944.3 kbp). In this region, we found 13 gene models that may be considered as potential candidates regulating the yield components (Table 5). Of those 13 candidate genes, nine are annotated as transmembrane transporters (GO:0055085) that are specialized in sugar transport (PF03083), two genes are involved in carbohydrate metabolism (GO:0005975), one gene is involved in lipid metabolism (ko00001), and one gene is involved in glucose metabolism (GO:0006011).

**Table 5:** Candidate genes identified within the main region on chromosome IV

Name	Start	End	Length (bp)	Strand	Description	Criollo v2
Thecc1EG016788t1	192,183	196,337	4154	+	Gluconokinase	Tc04v2_t000080.1
Thecc1EG016835t1	427,029	431,401	4372	−	Nodulin MtN21/EamA-like transporter family	Tc04v2_t000370.1
Thecc1EG016836t1	431,839	433,523	1684	−	Nodulin MtN21/EamA-like transporter family	Tc04v2_t000380.1
Thecc1EG016837t1	433,570	434,592	1022	−	Nodulin MtN21/EamA-like transporter family	Tc04v2_t000390.1
Thecc1EG016838t1	436,287	438,734	2447	−	Nodulin MtN21/EamA-like transporter family	Tc04v2_t000400.1
Thecc1EG016840t1	443,603	446,117	2514	−	Nodulin MtN21/EamA-like transporter family	Tc04v2_t000420.1
Thecc1EG016842t1	450,314	452,853	2539	−	Nodulin MtN21/EamA-like transporter family	Tc04v2_t000410.1
Thecc1EG016844t1	455,719	458,658	2939	−	Nodulin MtN21/EamA-like transporter family	Tc04v2_t000440.1
Thecc1EG016865t1	569,595	571,749	2154	+	Nodulin MtN3/SWEET family	Tc04v2_t000620.1
Thecc1EG016866t1	574,635	577,450	2815	+	Nodulin MtN3/SWEET family	Tc04v2_t000630.1
Thecc1EG016882t1	645,786	649,240	3454	−	GDSL-like Lipase/Acylhydrolase	Tc04v2_t000790.1
Thecc1EG016942t1	999,669	1,007,772	8103	+	Raffinose synthase family protein	Tc04v2_t001280.1
Thecc1EG016965t1	1,134,868	1,140,984	6116	+	ABC transporter B family member 26	Tc04v2_t001480.1

### Hierarchical clustering on principal components of the yield components

**Fig. 8:**
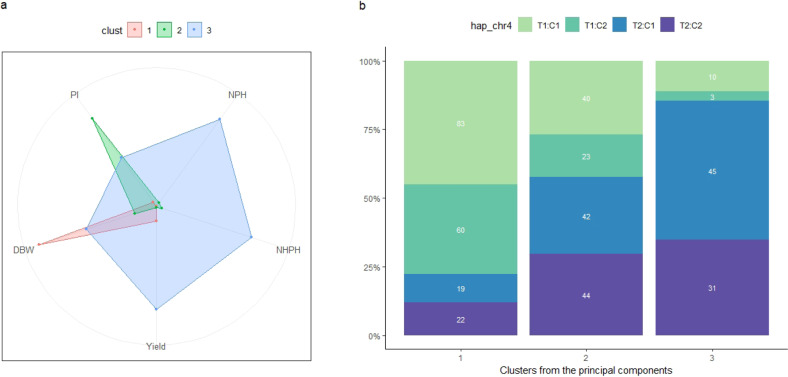
Phenotypical profile and haplotype frequency within each cluster identified after hierarchical clustering analysis on the principal components of the yield variables from the MP01 population. Panel (**a**) presents the radial charts for each cluster and five yield-related traits (DBW, NHP, NHPH, Yield, and PI), while panel (**b**) shows the frequency of the main haplotype on chromosome IV for each cluster

We applied the hierarchical clustering on those principal components^12^, to identify the groups of more productive trees (higher NPH, NHPH, and Yield) from MP01 population. The analysis identified three groups (clusters), of which DBW defines group 1, group 2 by PI and group 3 by the variables NPH, NHPH, and Yield (Fig. 8a and Supplementary Table 5). We provide the values for the cluster assignments for each individual of MP01 (Supplementary Table 5). From this analysis, group 3 contains the more productive trees from the MP01 population and may be used for a further selection of new higher-yielding varieties.

### Source of the alleles that increases the yield component and yield

To investigate the ancestry of the QTL with high LOD score on chromosome IV that increases the yield and yield components, we created a neighbor-joining tree of the ancestral haplotypes from a diversity panel that includes the parents of the F1 mapping population (‘TSH 1188’ and ‘CCN 51’), to identify the ancestry of the parental alleles (T1, T2, C1, and C2) associated with the cacao yield components on chromosome IV.

**Fig. 9:**
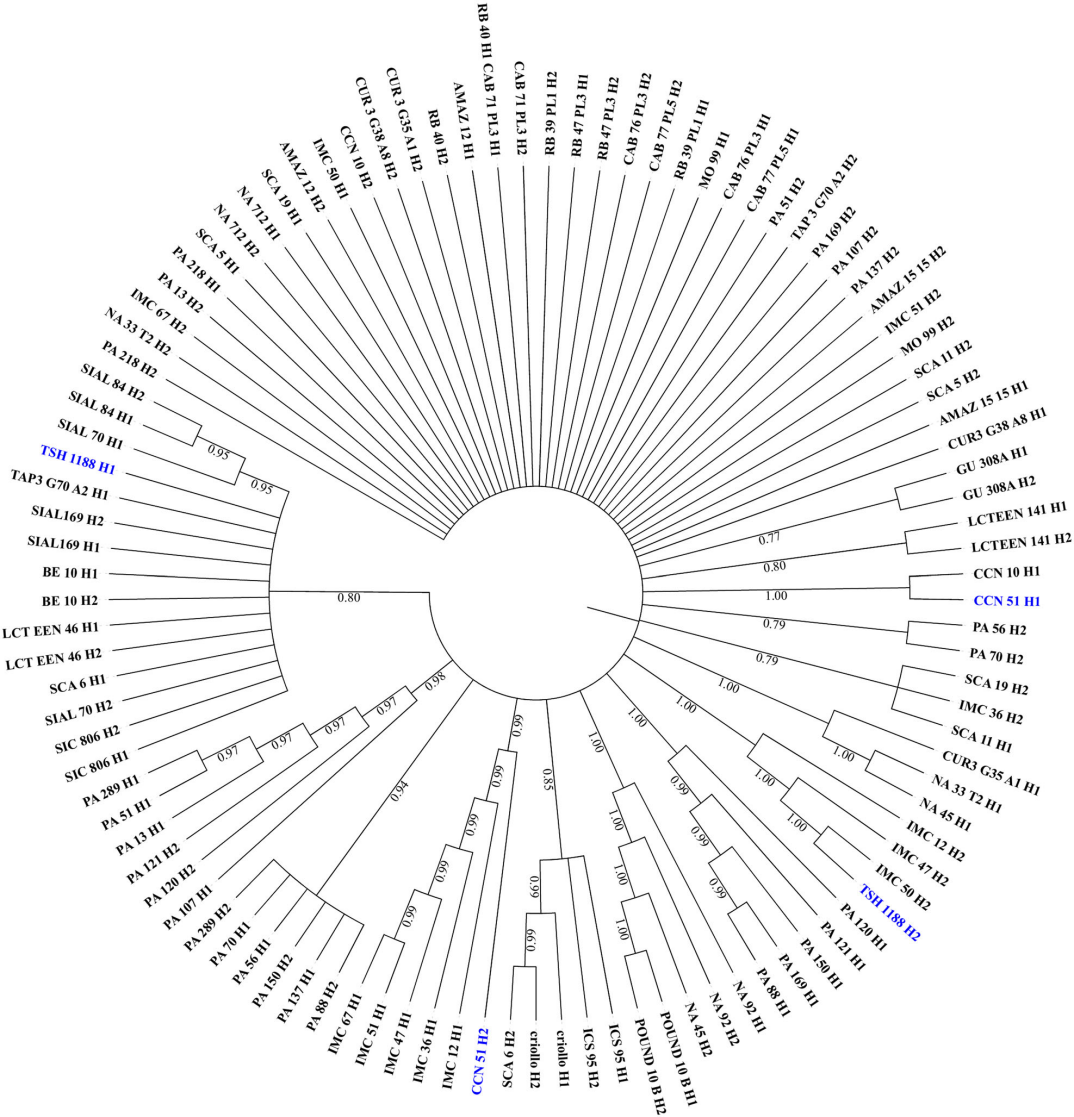
Neighbor-Joining tree analysis showing the origins of the haplotypes on the main QTL region on chromosome IV. Sixty-two markers in the QTL on chromosome IV, corresponding to 838.8 kb. The blue highlighted names are the first (H1) and second (H2) haplotype for each mapping population parent, ‘TSH 1188’ and ‘CCN 51’

The phylogenetic analysis of the main region on chromosome IV showed that ‘TSH 1188’ most likely inherited the haplotype T1 (AAG) from one of its great grandparents, ‘SCAVINA 6’ (SCA 6). In contrast, the second haplotype T2 (GGA) grouped with three varieties belonging to Iquitos genetic cluster, i.e., ‘IMC 12’, ‘IMC 47’, and ‘IMC 50’^13^. These results were expected, since another variety from Iquitos cluster^14^, ‘IMC 67’, is part of the ‘TSH 1188’ lineage^15,16^. In turn, the first paternal haplotype, C1 (AAA), grouped with the first haplotype of ‘CCN 10’. The second haplotype of ‘CCN 51’, C2 (GGG), grouped with varieties from Iquitos genetic group, including ‘IMC 67’, which is the paternal parent of ‘CCN 51’^14^ (Fig. 9). The haplotypes associated with increased yield in the mapping population, T2 (GGA) and C2 (GGG), are inherited from a member of the Iquitos genetic group.

## Discussion

### Correlation and multivariate analysis of the yield components

It is well known that plant productivity and crop yield rely on the translocation and partitioning of assimilated carbon (photoassimilates) and nutrients over the development of vegetative and harvestable organs^17^. Growing vegetative tissues and harvestable organs compete for the available assimilated carbon in the translocation stream. Competition for assimilated carbon has been shown in which changes in number of fruits (fruit load) affect fruit growth, fresh weight and carbohydrate concentration in *Malus domestica*^18^, *Citrus clementina*^19^, and *Actinidia Chinensis*^20^. In our study, we found a significant negative correlation between DBW with all the other variables, which indicates a competition for the assimilated carbon among those harvestable organs. The strongest correlation was between DBW and PI, which is essentially the number of pods required to obtain 1 kg of dry bean weight (Fig. 1). In quantitative terms, a heavier dry bean weight reflects in more photoassimilates allocated to the beans, which reduces the pod index. In this sense, PI is a key yield component that represents the extent of photoassimilate partitioning to the cacao beans. Therefore, it is an important variable to support the selection of high-yielding varieties.

The variables NPH, NHPH, and Yield, are more correlated with the first principal component (Dim.1) and, therefore, they are the most important in explaining the variability in the yield components from MP01. On the other hand, the variables PI and DBW are more correlated in the second (Dim.2) principal component (Fig. 2 and Supplementary Table 2). Therefore, combing the PCA eigenvalues from the first and second principal component would result in the selection of varieties that will be more productive but also have a lower PI.

### QTL mapping analysis

In our QTL mapping analysis, we found 24 genomic positions spread over eight chromosomes (no QTLs on chromosomes VII and X) associated with cacao yield components, and those regions may contain the primary candidate genes that control the phenotypic variation observed in MP01. Overall, most of the QTLs explained a low percentage of the variance, in which the highest values were for the markers on chromosome IV (from 4.1% to 23.60%), chromosome VIII (8.5%), chromosome III (5.7%), chromosome II (5.4%), and chromosome 6 (from 4.2% to 5.2%). Although the variance explained for most QTL was low, the total QTL variance explained per trait was large. The QTLs for NPH and NHPH explained 23.5% and 26.9% of the phenotypic variation, respectively. The variables DBW and PI showed the highest values, 60.0% and 31.4%, respectively. Finally, the variable Yield explained only 9.0% of the phenotypic variation. Among the traits evaluated, Yield showed the lowest value of heritability (H^2^ = 0.10) what demonstrated a high influence of the environment on the expression of this trait. The variability in rainfall may be one of the main causes of yield losses in rainfed farming systems^21^, such as in the area where the MP01 was grown. The yield components and yield are complex traits, and therefore, are controlled by multiple genes that can be affected by the different interactions with the environment, crop management, and growth and development processes. In our study, DBW and PI were the traits that showed the majority of QTLs with a lower contribution to the total percentage of variation explained. Those are quantitative traits, which are controlled by multiple genes involved in the physiological process that control seed size and weight^22^, and in the supply-demand balance of photoassimilates to sink organs^23^.

The actual phenotype that breeders are looking for commercial production is a tree that produces at least 50 pods per year, with a dry bean weight ranging from 1.0 to 2.0 g, such as ‘CCN 51’, the paternal parent of the MP01 population. ‘CCN 51’ is the most important variety in many countries in Central America and South America, with a potential production of at least 3 tons/ha. Since there are only two QTLs controlling NPH and NHPH, compared with the large number of QTLs found for the variable DBW, it will be more feasible to develop marker-assisted selection for trees producing higher number of pods. That is one of the reasons why we selected the regions on chromosome IV for discussion in this paper. The beginning of chromosome IV seems to be linked to many important traits. In our study, the region is associated with yield components. In other studies, the region from 1414 bp to 1,686,245 bp on chromosome IV is associated with other traits that may directly affect the cacao production, such as cherelle wilt ratio, fresh bean weight, fresh weight/pod, total number of pods, and disease resistance^24^, fatty acid composition^25^ and sexual-compatibility^26^. Another QTL associated with fat content in the MP01 population was also found on chromosome IV from 19,637,361 bp to 26,233,319 bp^25^. The top position of this QTL is located at the distance of 4,132,875 bp of one SNP mapped in our study for DBW, Tcm003s30366194. In this study, fat content represents 50.2–62.4% of the total dry weight of a bean^25^, therefore, it is also an important yield component. In our study, the most significant region was located from 0.11 to 5.16 cM of chromosome IV. This region harbored at least one significant SNP marker for each of the yield components evaluated and provided a significant level of contribution to the observed phenotypic variation (Table 2). Multiple QTL regions associated with cacao yield components were also mapped^2^ on chromosomes I, II, IV, V, and IX using phenotypic data from several years. In a second study, were found QTL regions at 72.3 cM on chromosome IV for traits such as bean length, shape index (the ratio of bean length to thickness), and fresh weight and the number of ovules per ovary^3^. Compared with our study, both QTL positions^2,3^ are closer to our second minor QTL located at 66.6 cM on chromosome IV for DBW (Table 2). In addition, the regions we mapped on chromosome II at 41.2 and 57.9 cM for DBW and PI are most likely the same regions mapped for bean traits (length, width, and thickness) and the number of ovules^3^. A significant region at the top of chromosome IV was also reported by Livingstone et al.^24^ for cherelle wilt, the total number of pods, and fresh bean weight. This QTL falls within one of the regions mapped for self-incompatibility^26^, which is a crucial yield factor^27^. The yield efficiency component, defined as the ratio of production to a cross-section of the trunk in kg cm^−3^, is significantly higher in self-compatible cacao varieties than in self-incompatible groups^28^. Those authors found a significant positive correlation between self-compatibility and yield efficiency, which also indicates a positive relationship between self-compatibility and final yield. Our findings demonstrate the importance of this region on chromosome IV, flanked by the markers Tcm004s00289192, Tcm004s00615809, Tcm004s01127580, to select high-yielding varieties.

### Main haplotypes increasing yield components and selection of candidate genes

Our analysis showed that the maternal haplotypes had the more significant effect on the phenotypical variation of the traits evaluated (Tables 3–4 and Figs. 4–7). So far in the MP01 population, the maternal alleles have been reported as the ones more affecting the economically important traits, such as disease resistance^11,29^, and fat content and fatty acid composition^25^. Likewise, our study showed that the alleles from ‘TSH 1188’ are the ones controlling the QTL regions with a higher LOD scores, such as the one found on chromosome IV. Only for the SNP markers Tcm002s23708704 and Tcm008s04113686 that the alleles from ‘CCN 51’ had a significant effect controlling the phenotypic variation of the yield components in our study. These results indicated a high maternal influence on the inheritance of complex traits in the MP01 population.

Given that we found a QTL explaining a large percentage of the phenotypic variation of the yield components, we can use the region on chromosome IV combined with haplotype effects on the other QTL regions, to select higher yield cacao varieties. A clear effect of the maternal haplotypes on the yield components was observed for the markers on the region on chromosome IV. In the MP01 population, the presence of the maternal haplotype T1 (AAG) was associated with an increase in dry bean weight. On the other hand, we observed a significant association of the haplotype T2 (GGA) with an increase in the number of pods and higher yield. These results are most likely reflecting the negative correlations found between the total number of pods and dry bean weight, as also observed between DBW and PI. Examination of MP01 trees that showed recombination events in this region confirmed that the inheritance of each maternal haplotype differentially influenced the dry bean weight (T1 = AAG) or the cacao yield (T2 = GGA). Besides, the frequencies of those haplotypes also changed according to the groups generated from hierarchical clustering on the principal components analysis (Fig. 8b). The frequency of T1:C1 and T1:C2 are higher on group 1 (cluster 1), which is defined by DBW, while decreased from group 2 (cluster 2) to 3 (cluster 3). However, the haplotype T2:C1 and T2:C2 are more frequent on group 3, which contains the more productive trees from MP01 population. Within this third group 85% of the trees possess the haplotype T2, being 50% for T2:C1 (GGA:AAA) and 35% for T2:C2 (GGA:GGG) (Fig. 8b). Those results point to the importance of haplotype T2 for selection of higher-yielding cacao varieties.

The trees displaying recombination events between the main markers on chromosome IV also allowed us to select candidate genes associated with cacao yield components. We identified the genes within a 944.1 kbp genomic region from 196,163 to 1,140,441 bp on chromosome IV. In this region, we identified candidate genes potentially involved in source-sink regulation and lipid metabolism. For instance, Thecc1EG016788t1 encodes a gluconate kinase, which is an essential enzyme of the oxidative pentose phosphate pathway^30^ that supplies NADPH during fatty acid synthesis in developing embryos^31^. Another possible lipid enzyme, Thecc1EG016882t1, may act in the breakdown of lipids and conversion to carbohydrates, and participate in the fatty acid metabolism during seed germination^32^. We also identified candidate genes belonging to this lipid enzyme family within recombination events near the markers on chromosomes II, V, VII, and IX (Supplementary Table 6).

A major group of nine candidate genes belong to the transmembrane solute carrier family were found located between 427,029 and 577,450 bp (150 kb) on chromosome IV, and twelve copies of the same group of genes are present within the QTL regions identified on chromosomes II, III, V and VI. The list with the names of the markers, marker position (bp), and the recombination interval and region size is provided in Supplementary Table 6. Such transmembrane carriers are crucial for multiple aspects of plant growth and development, particularly in plant responses to biotic and abiotic stresses^33^. Seven of those genes are annotated as MtN21/EamA-like transporters, which might play a significant role in the transport of amino acids^34^ and auxin^35^ throughout the whole plant. Those genes are within the recombination region close to the markers Tcm003s27977985 and Tcm003s30366194 on chromosome III (Supplementary Table 6). The transport of amino acids represents the main route to supply reduced nitrogen from source-to-sink tissues^36^. Besides, the growth and development of fruit and seeds require a stable supply of nitrogen for the production of storage proteins^37^. Finally, the supply of amino acids also controls biomass production and seed yield in *Pisum sativum* L.^6^.

Two other genes are part of the MtN3/SWEET gene family, which encodes a protein that principally acts like glucose, fructose, or sucrose transporters^38^, but it can also export plant essential micronutrients from source-to-sink organs^39^. Besides, we found the MtN3/SWEET genes within the QTL regions on chromosomes II (Tcm002s15300388) and III (Tcm003s00621672 and Tcm003s27977985) (Supplementary Table 6). The MtN3/SWEET proteins are crucial to phloem loading and soluble carbohydrate transportation during fruit development and seed filling in many crops^7,40,41^. Various SWEET genes participate in the partitioning of non-structural carbohydrates to the fruits in *M. domestica*^42^, and seed filling in *Oryza sativa* and *Zea mays* L.^43^. The genomic region where we identified copy-number variations of SWEET genes falls between the markers Tcm004s00289192 and Tcm004s00615809 on chromosome IV.

Two other candidate genes are located between markers Tcm004s00615809 and Tcm004s01127580. The gene model Thecc1EG016942t1 is a raffinose synthase (EC: 2.4.1.82). Members of the raffinose oligosaccharide family function as carbon reserve compounds required during seed maturation and protection against abiotic stresses^8^. The other *T. cacao* candidate gene, Thecc1EG016965t1, is a member of ATP-binding cassette transporter family (ABC transporter) that are associated with transportation of diverse metabolites^44^. Furthermore, those proteins transport fatty acids for lipid synthesis during the seed filling of *Arabidopsis thaliana*^45^ and are crucial during pollen development in *Ananas comosus*^9^.

Other candidate genes involved in synthesis and breakdown of lipid and carbohydrates were also found nearby the QTL regions on the other chromosomes identified in this study. The candidate genes annotated as phosphatidic acid phosphatase, non-specific phospholipase and beta-ketoacyl-[acyl-carrier-protein] synthase II (Supplementary Table 6) appear to affect the production and accumulation of storage lipid during seed development of *Jatropha curcas*^46^. A putative soluble inorganic pyrophosphatase and two putative plant invertase/pectin methylesterase inhibitors (Supplementary Table 6) may participate in starch and sucrose metabolism pathways^47^. All candidate genes flanked by the marker Tcm004s30466731 on chromosome IV seem to be involved in the synthesis of 1-aminocyclopropane-1-carboxylate oxidase homolog 1, which is a direct precursor of ethylene during fruit ripening in *Solanum lycopersicum*^48^. Likewise, the putative protein UDP-glucosyltransferase, found near the markers Tcm001s18406546 and Tcm008s00170802, also appears to be involved in fruit ripening^49^, and regulates secondary metabolites availability in peach^50^. Overall, the majority of the candidate genes identified within the QTL regions from our study may mediate important steps of lipid and carbohydrate metabolic pathways. Therefore, our study identified not only crucial candidate genes to be tested in functional gene expression studies but may also contribute in the development of molecular tools for improvement of cacao yield via breeding efforts.

### Conclusion

Here we report SNP-based QTL regions that are associated with different cacao yield components such as the total number of pods harvested, dry bean weight, yield, and pod index. The total number of healthy pods, yield, and pod index were the most important for the identification of the higher productive genotypes from the MP01 population. Then, those variables may be used for further selection of new varieties. The SNP markers associated with these yield components will be used to screen and to select high-yielding varieties via marker-assisted selection and genomic selection. Identification of the QTLs combined with the information from trees that showed recombination events in these QTL regions helped to identify candidate gene models affecting the phenotypic variation of yield components in cacao. Those candidate genes are not specific to the traits evaluated, because those genes may have multiples functions, but certainly they do contribute to the yield formation in other crops. Therefore, they are important to understand the yield formation in *T. cacao* as well. In other crops, such genes seem to play a significant role in source-sink transport of sugars and lipid metabolism. Therefore, those genes are the primary candidate to influence the preferential remobilization of carbohydrates, for instance, to set pods and the subsequent pod development and bean filling, which are economically essential components of cacao yield. In addition, the identification of haplotypes that contribute to either more pods per tree or to heavier bean weight per pod might help to select trees that are better fitting for the farmers in terms of labor, pest control, and management of the (post) harvest processes.

## Materials and methods

### MP01 segregating mapping population

The mapping population evaluated in this study, referred to as MP01, is part of the Mars cacao-breeding program located at the Mars Center for Cocoa Science (MCCS), Bahia, Brazil. MP01 comprises 459 trees from a cross between ‘TSH 1188’, used as the female parent, and ‘CCN 51’, used as the male parent. The female parent is related to Iquitos and Nanay genetic groups^13,24^, and the male parent has predominant ancestries from Iquitos, Criollo, and Amelonado^13,14,24^. Those parents contrast for many important traits, and the MP01 progenies segregate for disease resistance^11,29,51^, pod color^5^, fat content and fat composition^25^. The MP01 population also segregates for yield. ‘CCN 51’ is self-compatible^52,53^ and worldwide recognized as one of the most productivity cacao varieties with the capacity to reach 3 tons/ha in high productive farming system. ‘CCN 51’ also has a good level of cross-compatibility with many varieties, including ‘TSH 1188’. ‘TSH 1188’ is self-incompatible^53^, which results in lower pod production per tree and lower yield, compared with ‘CCN 51’. A study that evaluated the economically important traits showed that ‘TSH 1188’ had a dry weight of a single bean of 1.24 g and pod index ranging from 13 to 25^15^. ‘CCN 51’ had a dry weight of a single bean 1.62 g and pod index of 18. In terms of yield (dry bean weight in tons/hectare), ‘CCN 51’ showed values from 1034 to 1332 kg ha^−1^^16^. The evaluation of 20 trees in a clonal trial at MCCS from 2017 to 2019, demonstrated that we harvested three pods pods/tree/year for ‘TSH 1188’, while for ‘CCN 51’ we harvested 21 pods/tree/year. Overall, ‘CCN 51’ produced 0.539 kg/tree/year and ‘TSH 1188’ 0.074 kg/tree/year. Thus, the parents of the MP01 are also contrasting for yield components and yield, and their offspring segregate for those traits.

### Fresh to dry bean weight conversion factor

We counted and weighed the fresh beans (FBW) of five to ten pods of 100 progenies in MP01 to measure the fresh to dry bean weight conversion factor. We counted the beans and weighed the fresh beans for each pod. We then fermented the beans in polyethylene net bags for 7 days. After that, the beans were sun-dried to 7% moisture content as measured with a grain moisture and impurity analyzer (model G650, Gehaka, São Paulo, Brazil). The dry beans were weighed, and we found that the average dry weight was 0.36 of the fresh bean weight. After that, we used this ratio to estimate dry bean weight from fresh bean weight.

### Yield phenotypic data collection

The MP01 progeny dataset used in this study was collected from January 2007 through September 2018. Data were collected monthly on an individual tree basis. Per tree, we evaluated the total number of harvested pods (NPH), the number of healthy pods harvested (NHPH), which was calculated as the difference between NPH and the total pod lost by disease and pests. The total yield (Yield, kg/ha/year) was estimated 0.36 × FBW × 1111.11 (plant density, as number of trees per ha))/1000 for each year evaluated. The pod index (PI), which is defined as the number of pods required to obtain 1 kg of dry bean weight, was calculated as PI = NHPH × 1000/FBW × 0.36. The calculations were based on Mustiga et al.^54^, with modifications in fresh to dry bean weight conversation factor, as explained above. We also calculated the single dry bean weight (DBW), which was calculated as DBW = FBW × 0.36/total number of beans per pod. The DBW data were not calculated for 2009, 2010, and 2011 as FBW was not measured during those years.

### Data analysis

We calculated the best linear unbiased predictions (BLUP) from a linear mixed model fitted by maximum likelihood as described in Bates et al.^55^. The equation for the general linear mixed model fitted for each variable was:

$$y = \mu + X_{\mathrm{b}} + Z_{\mathrm{a}} + e$$

Where *y* is the vector of the response variable, *μ* is the variable mean, *a* is the random effect vector for individuals, *b* is the fixed-effect parameter for years, and *e* is the error. *X* and *Z* are incidence matrix values. Variance component estimates were used to calculate broad-sense heritability (H^2^). Also, we used BLUP values to carry out the principal components analysis for cacao yield components.

### Linkage map and QTL mapping

To perform the QTL mapping, we used a cacao linkage^11^ map containing 3526 SNPs and based on 459 trees from the MP01 population. For the initial detection of QTL with main effects, the first round of interval mapping (IM) was carried out to select SNP markers that significantly segregate for the traits evaluated from MP01. We used a regression approximation to maximum likelihood interval^56^ to estimate the QTL effects. The significant logarithm of odds (LOD) was determined by analyzing 10,000 permutations with *p*-values ≤0.05^57^. Afterward, the SNP markers showing the highest LOD values were selected as cofactors for multiple interval mapping (MQM) with MapQTL (version 6; Kyazma BV, Wageningen, the Netherlands)^58^. Graphical representations of chromosomes containing QTLs with significant effects and LOD score peaks were drawn using MapChart software, version 2.3^59^.

### Haplotype–phenotype associations

We used phased SNP haplotype data^60^ to identify the favorable haplotype–phenotype associations between yield and related variables. First, we calculate average data for each variable, and then we defined the phenotypic ranges using the frequency distribution function available at the R package ‘fdth’^61^, with the number of class intervals (k) set to four. The analysis applied Pearson’s chi-squared test for counting each allele combination within phenotypical classes for each variable evaluated, to test the significant haplotype–phenotype associations for each SNP marker with the highest LOD score. Parental haplotype/allele combinations were designated as T1 and T2 for ‘TSH 1188’, and C1 and C2 for ‘CCN 51’. We recorded the frequency of each parental haplotype and their combinations (T1C1, T1C2, T2C1, and T2C2) presented in each progeny of MP01.

### Candidate genes within the QTL regions

To identify potential candidate genes that drive the QTL effects and the phenotypic variations observed, we used the genomic region of the QTLs mapped in this study with the gene model annotation from Matina 1–6 v1.1^5^ and Criollo genome v2.0^4^. We explored the available gene model annotations in conjunction with the families, domains, and functional sites of proteins (INTERPRO), and other enrichment tools, such as biological pathways maps (KEGG) and enzymatic reactions (EC). We looked for the genes classified as involved in carbohydrate metabolism, e.g., membrane transport of sugars and amino acids, and synthesis and degradation of carbohydrates and starch. Besides, we searched for genes that might be involved in lipid metabolism, e.g., fatty acid biosynthesis and degradation.

### Phylogeny analysis of the main QTL region

We used the phased data of 106 SNP sequences from a diversity panel with 52 members from the ten *T. cacao* genetic groups^13^. Sixty-two SNP markers on the main QTL region on chromosome IV were used for this phasing. The markers go from Tcm004s00289192 to Tcm004s01127580, and correspond to a region of 838.8 kb. We used the neighbor-joining tree method^62^ to infer the phylogenetic relationship between diversity panel members. The percentage of replicate trees in which the associated taxa clustered together in the bootstrap test (1000 replicates) are shown next to the branches^63^. The tree is drawn to scale, with branch lengths in the same units as those of the evolutionary distances used to infer the phylogenetic tree. The distances were computed using the maximum composite likelihood method^64^. All positions containing gaps and missing data were eliminated (complete deletion option). The phylogeny was conducted in MEGA X^65^, and we edited the neighbor-joining tree according to Letunic and Bork^66^.

## Supplementary information

Supplementary Table S6- Candidate genes found in the QTLs regions associated with yield components on MP01 population

Supplementary Table S1 - Coordinates of the first two Principal components for MP01 individuals.

Supplementary Table S2 - Coordinates of the first two Principal components for yield components.

Supplementary Table S3 - The principal component eigenvalues and their percentage of variance.

Supplementary Table S4 - Description of each cluster by quantitative variables.

Supplementary Table S5 - Cluster assignments from hierarchical clustering on principal components.
